# Association of Serum GFAP with Functional and Neurocognitive Outcome in Sporadic Small Vessel Disease

**DOI:** 10.3390/biomedicines10081869

**Published:** 2022-08-02

**Authors:** André Huss, Ahmed Abdelhak, Benjamin Mayer, Hayrettin Tumani, Hans-Peter Müller, Katharina Althaus, Jan Kassubek, Markus Otto, Albert C. Ludolph, Deniz Yilmazer-Hanke, Hermann Neugebauer

**Affiliations:** 1 Department of Neurology, University Hospital Ulm, 89081 Ulm, Germany; andre.huss@uni-ulm.de (A.H.); hayrettin.tumani@uni-ulm.de (H.T.); hans-peter.mueller@uni-ulm.de (H.-P.M.); katharina.althaus@uni-ulm.de (K.A.); jan.kassubek@uni-ulm.de (J.K.); markus.otto@uk-halle.de (M.O.); albert.ludolph@rku.de (A.C.L.); 2 Department of Neurology, University of California San Francisco (UCSF), San Francisco, CA 94143, USA; ahmed.abdelhak@ucsf.edu; 3 Institute of Epidemiology and Medical Biometry, Ulm University, Schwabstraße 13, 89075 Ulm, Germany; benjamin.mayer@uni-ulm.de; 4 Department of Neurology, University Hospital Halle, 06120 Halle (Saale), Germany; 5 Clinical Neuroanatomy, Department of Neurology, Ulm University, 89075 Ulm, Germany; deniz.yilmazer-hanke@uni-ulm.de; 6 Department of Neurology, University Hospital Würzburg, 97080 Würzburg, Germany

**Keywords:** chitinase-3-like protein 1, GFAP, neurofilaments, white matter hyperintensities, biomarker, CSVD

## Abstract

Cerebrospinal fluid (CSF) and serum biomarkers are critical for clinical decision making in neurological diseases. In cerebral small vessel disease (CSVD), white matter hyperintensities (WMH) are an important neuroimaging biomarker, but more blood-based biomarkers capturing different aspects of CSVD pathology are needed. In 42 sporadic CSVD patients, we prospectively analysed WMH on magnetic resonance imaging (MRI) and the biomarkers neurofilament light chain (NfL), glial fibrillary acidic protein (GFAP), chitinase3-like protein 1 (CHI3L1), Tau and Aβ1-42 in CSF and NfL and GFAP in serum. GFAP and CHI3L1 expression was studied in post-mortem brain tissue in additional cases. CSVD cases with higher serum NfL and GFAP levels had a higher modified Rankin Scale (mRS) and NIHSS score and lower CSF Aβ1-42 levels, whereas the CSF NfL and CHI3L1 levels were positively correlated with the WMH load. Moreover, the serum GFAP levels significantly correlated with the neurocognitive functions. Pathological analyses in CSVD revealed a high density of GFAP-immunoreactive fibrillary astrocytic processes in the periventricular white matter and clusters of CHI3L1-immunoreactive astrocytes in the basal ganglia and thalamus. Thus, besides NfL, serum GFAP is a highly promising fluid biomarker of sporadic CSVD, because it does not only correlate with the clinical severity but also correlates with the cognitive function in patients.

## 1. Introduction

Cerebral small vessel disease (CVSD) is the cause of one-fourth of ischemic strokes and a major contributor to cognitive decline and functional loss in the elderly [1]. The main driver for pathophysiological changes and the clinical presentation in CSVD is damage to the small arterioles in deep white matter. The current evidence points especially to vascular endotheliopathy, which results in substantial damage to the blood–brain barrier with a thickening of the arteriolar vessel walls, ultimately leading to vessel obliteration. Subsequent changes in the surrounding brain parenchyma induced by tissue hypoxia and cytokine influx from blood include activation of the microglia and astrogliosis and neuroaxonal damage [1,2,3,4,5].

Magnetic resonance imaging (MRI) hallmarks of CVSD encompass small subcortical infarcts, lacunes, white matter hyperintensities (WMH), dilatation of perivascular spaces, microbleeds and brain atrophy [1,5,6]. The classification of CSVD severity and clinical relevance mainly depends on the location and volume of WMH [7,8] and is commonly graded according to the Fazekas visual rating scale [9]. The Fazekas scale shows good intra- and interrater reliability in cross-sectional studies and correlates closely with more sophisticated volumetry [10]. However, the association of MRI pathology and functional outcome such as cognition is moderate at best [4,11,12,13]. Therefore, the establishment of body fluid biomarkers, preferentially in the serum, that are reliable indicators for the progression and dynamics of CSVD is highly desirable.

Neurofilament light chain (NfL) is a well-characterised marker for axonal damage in neurological diseases [14]. NfL was shown to be higher in the cerebrospinal fluid (CSF) of patients with severe white matter lesions (WML) compared to patients with mild or moderate WML [15]. Elevation of the CSF NfL levels further reflects an increasing degree of white matter changes [16] and is associated with the imaging marker of CSVD and future cognitive impairment in various domains [12,13]. Moreover, the NfL levels in serum are higher in patients with ischemic stroke, recurrent ischemic stroke and recent small subcortical infarct compared to the controls [3,17]. The phosphorylated heavy chain of neurofilaments (pNfH) is another potential marker of axonal damage after acute ischemic stroke [18]. Astrocytic activation and reactive gliosis may be another promising target for stroke treatment [19] and for the capturing and monitoring of CSVD severity, which was studied using two well-established biomarkers of alterations in the astroglial somata and processes—namely, glial fibrillary acidic protein (GFAP) and Chitinase-3-like protein 1 (CHI3L1). GFAP is an intermediate filament protein that was shown to be elevated in the CSF of acute stroke patients [20,21]. CHI3L1 is also known to be expressed in various other neurological diseases under neuroinflammatory conditions [22], and the deletion of CHI3L1 was shown to accelerate strokes in mice [23].

To the best of our knowledge, however, these highly encouraging stroke biomarkers have never been analysed together in CSVD patients to directly compare their diagnostic or prognostic values. While the GFAP and CHI3L1 levels are known to be increased in strokes, and the GFAP elevation may help differentiate intracranial haemorrhage from ischemic stroke [6,20,21,22,23], the correlation of the two astroglial markers with CSVD severity and other CSVD markers remains uncertain. The analysis in our prospective CSVD patient cohort therefore aimed at characterising a panel of CSF and serum biomarkers that included GFAP and CHI3L1 in correlation with clinical and imaging markers of disease severity. Furthermore, tissue from disjoint autopsy cases with CSVD was examined to evaluate the extent and topographical distribution of GFAP- and CHI3L1-immunoreactive cells in the brain and to identify the source of increased biomarker levels in the serum of the clinical cohort.

## 2. Materials and Methods

### 2.1. Patient Selection and Inclusion

In this study, patients with sporadic CSVD-related stroke and vascular cognitive impairment were prospectively recruited for the study at the stroke unit and the memory outpatient clinics of the University Hospital of Ulm, Germany, between 2015 and 2017. Patients with MRI-confirmed recent small subcortical lacunar infarction, MRI evidence of WMH suggestive of underlying CSVD and cognitive impairment on a cognitive screening study were considered for inclusion to the study. For the diagnosis of lacunar infarction and white matter hyperintensities, we applied the STandards for ReportIng Vascular changes on nEuroimaging (STRIVE) criteria [1].

Sporadic CSVD-related stroke was diagnosed after the exclusion of other stroke aetiologies by thorough cerebrovascular workup, including duplex sonography or computed tomography (CT)–angiography of brain-supplying arteries, transthoracic echocardiography and transoesophageal echocardiography if indicated, continuous electrocardiography (ECG) monitoring for at least 72 h and additional Holter ECG in high-risk patients for atrial fibrillation (i.e., enlarged left atrium and high rate of supraventricular extra systoles during continuous monitoring), routine blood tests and past medical history negative or not suggestive of hereditary cerebrovascular diseases.

Patients discharged from our stroke unit were followed up three months later in our outpatient clinic and included in our study if cognitive impairment persisted at this time point and delirium was ruled out as a cause. During this visit, degenerative dementias—in particular, Alzheimer’s disease (AD), as well as other causes of cognitive impairment such as metabolic and endocrinological disorders—were ruled out by extensive neuropsychological examinations and cognitive testing using the CERAD-Plus test battery and by extended laboratory investigations in blood and CSF obtained through lumbar puncture. Based on our in-house reference ranges, the CSF levels were considered suggestive for Amyloid pathology when the Amyloid 1–42 values were below 600 pg/mL and indicative for neurodegeneration when the Tau values were higher than 400 pg/mL.

Each patient’s history was reviewed, in particular with regard to previous antihypertensive treatment and pre-existing retinal disease, the blood pressure was measured and the National Institute for Health Stroke Scale (NIHSS) and the modified Rankin Scale (mRS) were assessed by a trained rater [24,25]. Patients’ characteristics can be found in Table 1.

Patients admitted to the Neurology Department, including those treated in the outpatient clinics, were approached for consent to autopsy. Tissue from autopsy cases was preserved at the Ulm University Tissue Bank for routine neuropathological examination and further investigations. All studies were conducted in compliance with the University Ethics Committee guidelines, as well as German federal and state laws governing human tissue usage and in accordance with the Declaration of Helsinki. Informed written permission was obtained from all patients and/or their next of kin for autopsy.

### 2.2. Magnetic Resonance Imaging (MRI)

MRI data were acquired using a 1.5 T scanner (Symphony, Siemens Medical, Erlangen, Germany) to quantify the WMH. Altogether 42 T2-weighted coronal slices (Fluid Attenuated Inversion Recovery/FLAIR, TR/TE 8500/82 ms) of 3.0 mm in thickness, 0.43 × 0.43 mm^2^ in-plane resolution and 512 × 448 voxels matrix dimension were scanned. Neuroimaging findings of CSVD (lacunes, cerebral microbleeds and perivascular spaces) were defined according to the STandards for ReportIng Vascular changes on nEuroimaging (STRIVE), and WMH were graded according to the Fazekas visual rating scale [1,26,27]. In addition, the WMH volume was quantified to determine the severity of CSVD.

**Figure 1:**
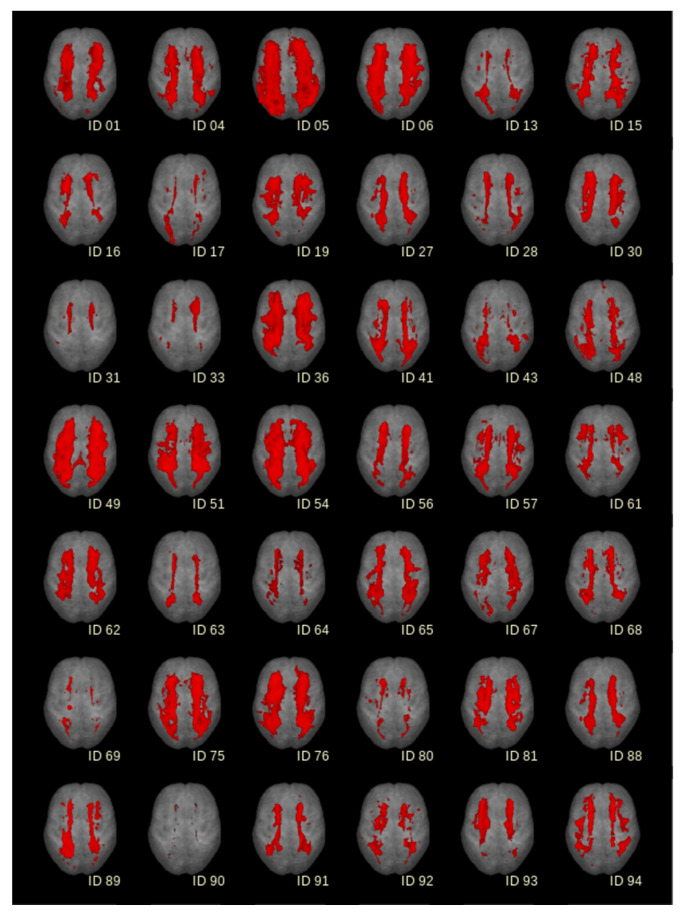
White matter hyperintensities (WMH) for 42 patients detected from FLAIR-MRI recordings in axial projection views. For comparability, individual results were normalised to the Montreal Neurological Institute (MNI) coordinate frame and displayed on the same morphological background (segmented averaged brain).

T2-weighted images were scored visually according to the Fazekas scale and additionally analysed by an in-house software based on a semi-automatic image intensity analysis to calculate the volume of white matter hyperintensities (WMH) [28,29,30]. To identify lesion-related voxels, voxels within an operator-defined intensity range were identified slice-wise. The thresholds for selecting WMH voxels had to be adjusted operator-dependently, as T2-weighted MR images intrinsically represent no absolute intensity values. The number of detected voxels (total amount of voxels corresponds to the total volume of the lesion) was identified as the WMH. The WMH for the 42 individuals has been summarised in Figure 1 as axial projection views. According to the STRIVE definitions [1], we defined a cerebral microbleed as a focal area (<10 mm) of low signal intensity on the gradient echo. Lacunes were defined and counted as ovoid or round, fluid-filled CSF-cavities in the subcortical areas, with a diameter between 3 and 15 mm and surrounded by a hyperintense rim on FLAIR sequences. Visible Virchow-Robin perivascular spaces were identified as linear and round or ovoid spaces following the course of a vessel with a perpendicular diameter <3 mm and without a hyperintense rim on FLAIR imaging.

### 2.3. Lumbar Puncture

Lumbar puncture (LP) was performed for diagnostic purposes only with written informed consent from all patients. CSF and serum samples were collected on the same day and stored according to the consensus protocol for the standardisation of CSF collection and biobanking [31]. Blood-contaminated CSF samples were excluded.

### 2.4. CSF and Serum Biomarker Analyses

Neurofilament light chain (NfL) in the CSF was analysed using the ELISA kit from Uman Diagnostics (UmanDiagnostics AB, Umeå, Sweden) and in the serum with a single-molecule array (Simoa^®^, Quanterix Corporation, Lexington, MA, USA). Neurofilament heavy chain (pNfH) was quantified with Phosphorylated Neurofilament H Human ELISA (Biovendor, Heidelberg, Germany). GFAP in the CSF and serum was analysed using Simoa^®^ (Quanterix Corporation, Lexington, MA, USA). Chitinase-3-like protein 1 (CHI3L1) in the CSF was analysed using the Human Chitinase 3-like 1 Quantikine ELISA Kit (R&D Systems, Minneapolis, MN, USA). Aβ1-42 and Tau in the CSF were analysed using Innotest^®^ and Tau-ELISA, respectively (Fujirebio Germany GmbH, Hanover, Germany). All analyses were performed according to the manufacture’s manual, and samples were run in duplicates. Measurements were interpreted as valid if duplicate measurements showed a coefficient of variation below 15%.

### 2.5. Cognitive Testing

The mini mental state examination and the expanded Consortium to Establish a Registry for Alzheimer’s disease Neuropsychological Assessment Battery (CERAD-NAB Plus) were applied to assess the cognitive function in our cohort. Beside the standard nine variants in the CERAD-NAB [32], the CERAD-NAB Plus was modified by adding three tests of executive functioning and mental speed (Trail Making Tests A and B and S-Words) to increase its sensitivity toward subcortical deficits, which are common in vascular dementia [33]. The S-Words (phonemic fluency) test is a task measuring the ability of participants to generate as many words as possible beginning with the given letter ‘S’ within 60 s. In TMTA, which is a measure of psychomotor speed and visual scanning, participants must draw lines connecting the numbers 1 through 25 in ascending order as quickly as possible. The numbers are distributed over a sheet of paper. Performance is evaluated using the total completion time. Executive functioning was evaluated using TMTB, a measure of mental flexibility and the ability to switch between two different modalities. In TMTB, both numbers (1–13) and letters (A–L) are distributed over a sheet of paper. Participants are asked to draw lines to connect numbers and letters in ascending pattern, but with the added task of alternating between modalities (i.e., 1–A–2–B–3–C, etc.). Again, performance was evaluated using the total completion time in seconds. All test results were inherently corrected for age, sex and education and expressed as z-values.

### 2.6. Immunohistochemistry

GFAP and CHI3L1 expression was studied in the brain of 7 CSVD and 5 control cases without neurological disorders (Table 2). The brains of all autopsied subjects were fixed in a 4% formaldehyde solution and underwent a routine neuropathological examination, as described previously [34,35]. A coronal mid-hemispheric block (approx. 1 cm thick) was embedded in polyethylene glycol (PEG 1000, Merck, Carl Roth Ltd., Karlsruhe, Germany). Multiple 100-µm-thick consecutive sections were obtained from each block with the aid of a sliding microtome (Jung, Heidelberg, Germany). Immunohistochemistry (IHC) was performed with primary antibodies against GFAP (1:1000, monoclonal mouse, Abcam, Cambridge, UK) or CHI3L1 (1:750, polyclonal goat, R&D Systems, Exton, PA, USA) and a secondary biotinylated antibody (1:200; 2 h, room temperature, Vector Laboratories, Burlingame, CA, USA). The immunohistochemical reaction was visualised with an avidin–biotin–peroxidase complex (ABC Vectastain Kit, Vector Laboratories, Burlingame, CA, USA) and the chromogen 3,3′-diaminobenzidine tetrahydrochloride (DAB; Sigma Taufkirchen, Germany), which was intensified using Cobalt(II)Chloride (32 nM) for the labelling of CHI3L1. Omission of the primary antibody resulted in a lack of staining.

### 2.7. Quantitative Histopathological Analyses

For analyses of periventricular GFAP immunoreactivity, histological mid-hemisphere sections were scanned in the presence of a scale bar at 300 dpi resolution (HP ScanJet Pro 2500 FL scanner). Images were opened with the software ImageJ, version 1.52n (National Institutes of Health, Bethesda, MD, USA, http://imagej.nih.gov/ij, accessed on 26 July 2022). The scale bar within each image was traced using the free hand straight line tool for setting the scale. After successful image calibration, the GFAP-immunoreactive area around the central part of the lateral ventricle was delineated with the free hand polygon tool. Next, the circumscribed GFAP-immunoreactive area was measured (‘Measure’ function under the ‘Analyze’ menu), and the results were exported. For analyses of the CHI3L1 pathology, the boundaries of the periventricular zone were marked on the immunostained sections at a 10-mm distance from the ependymal layer of the central part of the lateral ventricle. The marked periventricular zone was screened for CHI3L1-immunoreactive glial cell clusters with an upright bright field microscope using the 10× objective (AX10 microscope, Zeiss, Jena, Germany), and the number of clusters encountered was recorded.

### 2.8. Statistical Analyses

All statistical tests were performed using SPSS^®^ Statistics version 25 (IBM Corporation, Armonk, NY, USA), and the figures were created with GraphPad Prism 8 software (GraphPad Software Inc., La Jolla, CA, USA). The Shapiro–Wilk test was used to examine the distribution of the data. Accordingly, Kruskal–Wallis with Dunn’s multiple comparison test was used to compare medians in skewed distributed datasets. An ordinal regression analysis was performed to detect the potential influence of the covariates (e.g., age and sex). Spearman’s rank correlation was used to measure the correlations. The Mann–Whitney *U* test was used for histopathological comparisons of the CSVD and control cases. A two-tailed *p*-value ≤ 0.05 was considered statistically significant.

## 3. Results

### 3.1. Patients’ Characteristics

A total of 42 patients with sporadic CSVD with a recent stroke and vascular cognitive impairment were included in this study during 2015 and 2017. The patients’ characteristics can be found in Table 1. For all included patients, Aβ1-42, Tau, NfL, GFAP and CHI3L1 were analysed in the CSF, and NfL and GFAP were measured in the serum. The levels were compared to the extent of WMH according to Fazekas [9], i.e., mild, moderate and severe, to clinical scores (NIHSS and mRS) and were correlated to cognitive functions (assessed by MMST, Trail Making Tests A and B and S-Words). The MRI for the rating of the WMH of all 42 patients are shown in Figure 1.

**Table 1:** Patients’ characteristics. CSVD = cerebral small vessel disease; IQR = interquartile range; mRS = modified ranking score; NIHSS = NIH stroke scale; WMH = white matter hyperintensities.

	All Patients = 42			*p*-Value
CSVD degree	1 (mild)	2 (moderate)	3 (severe)	-
Number (n)	8	18	16	-
Male/Female	7/1	10/8	9/7	0.22
Age (range)	71 (64–80)	77 (48–89)	74 (61–91)	0.25
mRS (IQR)	1.0 (0.0–1.75)	1.5 (1.0–3.0)	2.0 (1.0–3.0)	0.06
NIHSS (IQR)	0.5 (0.0–1.75)	1.0 (0.0–2.0)	3.0 (1.0–4.0)	0.07
WMH volume (IQR)	8.8 (3.8–11.2)	26.6 (20.5–30.7)	60.1 (45.2–88.0)	<0.0001

**Figure 2:**
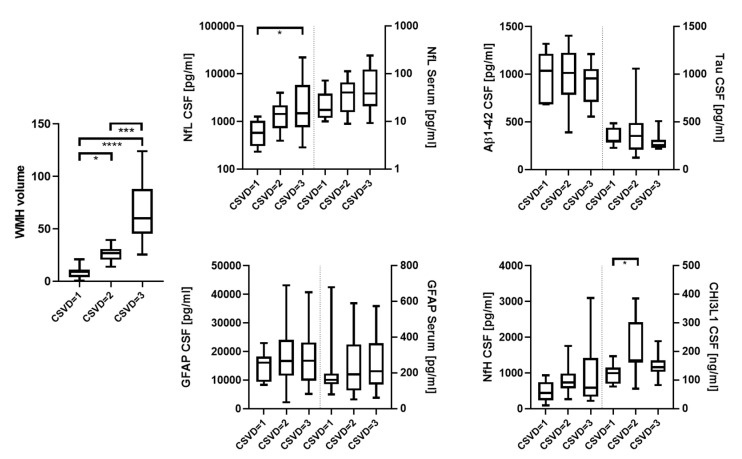
Degree of CSVD based on Fazekas (mild, moderate and severe) compared to WMH, NfL (CSF and serum), GFAP (CSF and serum), Aβ1-42 (CSF), Tau (CSF), NfH (CSF) and CHI3L1 (CSF). * = *p* < 0.05; *** = *p* < 0.001; **** = *p* < 0.0001.

**Figure 3:**
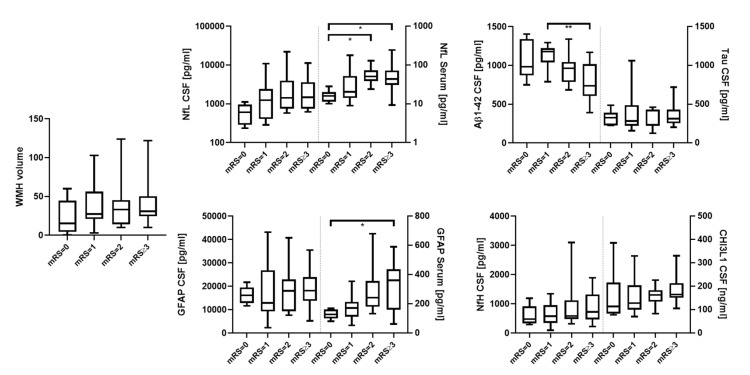
mRS (0, 1, 2 and ≥3) compared to WMH, NfL (CSF and serum), GFAP (CSF and serum), Aβ1-42 (CSF), Tau (CSF), NfH (CSF) and CHI3L1 (CSF). * = *p* < 0.05; ** = *p* < 0.01.

**Figure 4:**
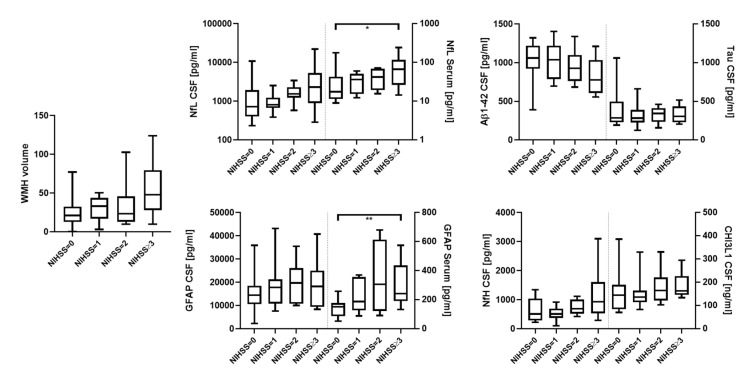
NIHSS (0, 1, 2 and ≥3) compared to WMH, NfL (CSF and serum), GFAP (CSF and serum), Aβ1-42 (CSF), Tau (CSF), NfH (CSF) and CHI3L1 (CSF). * = *p* < 0.05; ** = *p* < 0.01.

**Figure 5:**
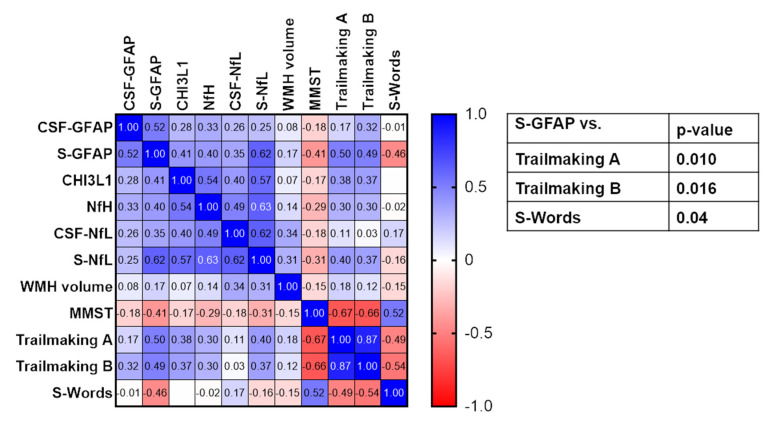
Correlation matrix of CSF and serum (S) biomarkers and neurocognitive parameters (MMST, Trail Making A and B and S-Words). Numbers in the heat map give the Spearman’s correlation coefficient. *p*-values in the table only show significant correlations after correction for multiple testing with Bonferroni’s correction.

The CSF levels of NfL and CHI3L1 were significantly increased for the WMH scores based on Fazekas [9] when comparing patients with mild and severe (580 vs. 1494 pg/mL. *p* = 0.022, Figure 2) and mild and moderate Fazekas scores (124.3 ng/mL vs. 169.7 ng/mL, *p* = 0.013), respectively (Figure 2). CSVD severity assessed with mRS and NIHSS was also associated with significantly higher levels of serum NfL (mRS = 0 vs. 2 and ≥3, 16.0 pg/mL vs. 50.9 pg/mL and 43.5 pg/mL, *p* = 0.035 and 0.028, respectively; NIHSS = 0 vs. ≥3, 17.6 pg/mL vs. 66.5 pg/mL, *p* = 0.033, Figure 3 and Figure 4), as well as serum GFAP (mRS = 0 vs. ≥3, 127.7 pg/mL vs. 361.8 pg/mL, *p* = 0.016; NIHSS = 0 vs. ≥3, 150.8 pg/mL vs. 241.4 pg/mL, *p* = 0.006, Figure 3 and Figure 4). In contrast, CSF Aβ1-42 were reduced in CSVD patients with higher mRS scores (mRS = 1 vs. ≥3, 1179 pg/mL vs. 737 pg/mL, *p* = 0.001, Figure 3 and Figure 4). Finally, and most importantly, we were only able to see significant correlations between serum GFAP and neurocognitive functions (Trail Making Test A: ρ = 0.50 and *p* = 0.010, Trail Making Test B: ρ = 0.49 and *p* = 0.016 and S-Words: ρ = −0.46 and *p* = 0.04, Figure 5).

These main findings remained significant after correcting for age, gender, BMI and renal function.

### 3.2. Histopathology

**Figure 6:**
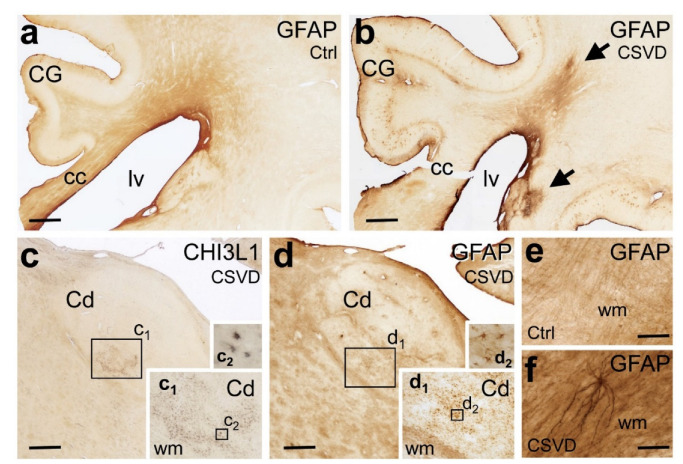
Immunohistochemical demonstration of the astroglial markers glial acid fibrillary protein (GFAP) and chitinase 3-like 1 (CHI3L1) in 100-µm-thick mid-hemisphere sections in CSVD and the controls. (**a**,**b**) CSVD case (**b**; Case # 1) with MRI-visible white matter hyperintensities (WMH) displays stronger GFAP immunoreactivity in the white matter (arrows) than the case with acute stroke but only minor subcortical CSVD and no WMH (**a**; Case # 12). (**c**,**d**) CSVD case (**c**,**d**; Case # 7) with clusters of CHI3L1-positive astrocytes in the caudate nucleus (Cd) in the absence of immunoreactive astrocytes in the neighbouring white matter. The density of GFAP-expressing reactive astrocytes is also high in the Cd, whereas the surrounding white matter tracts contain bundles of GFAP-positive fibrillary astrocytic processes. Boxed areas in (**c**) with CHI3L1-positive glial cell clusters and in (**d**) with GFAP-positive glial cell clusters are shown at higher magnification in the insets (**c1**,**c2**) and in the insets (**d1**,**d2**), respectively. (**e**,**f**) At higher magnification, the periventricular white matter of CSVD cases (**f**; Case # 5) presents with a much higher density of GFAP-positive fibrillary astrocytic processes than controls (**e**; Case # 9). Cc: corpus callosum, Cd: caudate nucleus, CG: cingulate gyrus, CHI3L1: chitinase-3-like protein 1, CSVD: cerebral small vessel disease, Ctrl: control, GFAP: glial fibrillary acidic protein, lv: lateral ventricle and WMH: white matter hyperintensities. Scale bars: 1000 µm (**a**–**d**) and 100 µm (**e**,**f**).

Autopsied cases included to the study had Alzheimer’s-related argyrophilic neurofibrillary tangle (NFT) stages 3 or less (except for an 89-year-old female at stage 4), as well as varying degrees of extracellular Aβ deposition at cortical amyloid stages 0–C [36]. None of the cases were diagnosed with other tauopathies or with alpha-synucleinopathy (Table 2). The periventricular white matter of CSVD cases showed a much higher density of astrocytes with fibrillary GFAP-immunoreactive processes than the controls. While clusters of CHI3L1-positive astrocytes could be also found in this zone, the density of these cells was much lower in the white matter compared to the neighbouring subcortical nuclei, and the differences between the CSVD and control groups were less prominent (Figure 6 and Table 2). In CSVD cases, the basal ganglia and thalamic nuclei also harboured numerous GFAP- and CHI3L1-positive astrocytes, which formed detached or confluent cell clusters at corresponding regions of consecutive brain sections (Figure 6).

**Table 2:** GFAP and CHI3L1 immunoreactivity in the periventricular white matter in CSVD cases and the controls. Quantitative histopathological analyses of the white matter (wm) area with enhanced GFAP expression (area measured in cm^2^) and with CHI3L1-positive cell clusters (number within a 10-mm radius) around the central part of the lateral ventricle at the mid-hemispheric level. In CSVD cases, the medial periventricular zone (PVM) with strong GFAP expression extends much further into the lateral periventricular zone (PVL), with moderate enhancement of the GFAP expression when compared to the controls. Moreover, enhanced GFAP expression in the PVL of some CSVD cases extends farther to the deep white matter, often reaching the cortex of the superior frontal gyrus (§). ACA = anterior cerebral artery; Art. = arterial; Ctrl = control; CRF = chronic renal failure; CSVD = cerebral small vessel disease; f = female; m = male, L = left; MCA = middle cerebral artery; mm = millimetre; PCA = posterior cerebral artery; PV = periventricular region; PVL = lateral periventricular region; PVM medial periventricular region; R = right; Staging AD/Aβ = staging for Alzheimer-related neurofibrillary tangles/Aβ deposits [24]; TIA = transient ischaemic attack; wm = white matter. * *p* < 0.05, ** *p* ≤ 0.01.

No	Age	Sex	Group	Diagnosis	Staging AD/Aβ	GFAP-PVM wm (cm^2^)	GFAP-PVL wm (cm^2^)	GFAP-PV Total wm (cm^2^)	CHI3L1-PV wm (# Cell Cluster)
1	89	f	CSVD	Art. hypertension, mixed dementia, TIA, aneurysm ACA, bradycardiac arrhythmia with decompensated CRF	IV/C	0.240	0.535	0.775	0
2	79	m	CSVD	Art. hypertension, aortic valve replacement	I/B	0.263	1.167 ^§^	1.430 ^§^	1
3	77	m	CSVD	Pulmonary embolism	0/0	0.120	0.649	0.769	5
4	57	m	CSVD	Renal cancer	0/0	0.223	0.740	0.963	0
5	81	f	CSVD	(Sub)acute ischemic MCA infarct L	I/0	0.638	2.172 ^§^	2.810 ^§^	1
6	60	f	CSVD	Pontine bleeding	I/A	0.357	0.749	1.106	0
7	57	f	CSVD	Breast cancer, infarction in internal capsule	I/A	0.490	1.890 ^§^	2.380 ^§^	3
8	78	f	Ctrl	Pulmonary embolism, renal failure	0/0	0.134	0.157	0.291	0
9	71	f	Ctrl	Left heart failure	0/0	0.125	0.538	0.663	0
10	67	f	Ctrl	Ruptured aortic aneurysm with bleeding and circulatory arrest	0/0	0.220	0.211	0.431	0
11	54	f	Ctrl	Ovarian cancer	I/B	0.100	0.777	0.877	0
12	84	f	Ctrl	Subacute cerebral MCA and PCA infarctions R	II/0	0.181	0.341	0.522	1
Mean					CSVD	0.333	1.129	1.462	1.429
					Ctrl	0.152	0.405	0.557	0.200
S.E.M.					CSVD	0.067	0.246	0.308	0.719
					Ctrl	0.021	0.114	0.100	0.200
Mann–Whitney *U* test									
				Mann–Whitney *U*		4.000	5.000	2.000	10.000
				standardised test statistic		−2.192	−2.030	−2.517	−1.370
				exact *p*-value (2-sided)		0.030 *	0.048 *	0.010 **	0.268

## 4. Discussion

In our prospectively collected hospital-based cohort of sporadic CSVD patients, we studied both the CSF and serum profiles of established and innovative biomarkers. Here, we were able to show that CSF-NfL is elevated with an increased severity of CSVD, which is in agreement with the previous findings [12,13,15]. The involvement of astroglial processes was also indicated by higher CSF-CHI3L1 levels in patients with moderate CSVD compared to mild CSVD cases. Of note, especially the serum levels of NfL and GFAP showed good correlation with a more severe clinical disease course based on mRS and NIHSS. We were not able to find consistent differences here for any other marker, including the WMH volume. Even though one might have expected a better detection of markers of axonal damage and glial activation in the CSF than in the serum, a high sensitivity of serum markers to disease severity was indeed shown in various neurological diseases for NfL and GFAP. Serum NfL was shown to be highly sensitive to the activity of CSVD and ischemic stroke [3,13,17]. Moreover, NfL was also detected in brain-derived circulating extracellular vesicles (EVs) in the serum of mice in the bilateral carotid artery occlusion model, as well as in EVs isolated from the human prefrontal cortex of mixed Alzheimer’s disease cases with cerebrovascular lesions, including CSVD and cortical microinfarcts [37]. However, the serum GFAP levels had not been examined yet in CSVD. So far, the serum GFAP levels were only shown to be a potential biomarker in intracerebral haemorrhage and ischemic stroke [21,38] and to correlate with the disease severity in multiple sclerosis [39,40,41], Alzheimer’s disease [42] and NMOSD [43,44]. A striking observation in our study was a moderate and significant correlation of serum GFAP with cognitive functions in the Trail Making Tests A and B and S-Words, all of which are specifically sensitive toward the pathognomonic subcortical deficits of CSVD. These findings may indicate the potentially important and primary role of astrocytic dysfunction in the outcome of CSVD, in addition to concurrent axonal damage. This idea fits well with pathoanatomical considerations, since astrocytes are an integral part of the neurovascular unit in close proximity to the endothelium, which is hit first and hardest by microangiopathic processes of CSVD [45]. In contrast, NfL may appear with a delay and to a lesser extent after disease progression also leads to axonal degeneration. At present, there is no established CSF or serum biomarker to monitor and predict the functional and cognitive outcome in CSVD [2,46], whereas the extent and volume of WMH offer only weak-to-moderate correlations at best [12,13]. The potential impact of serum GFAP and NfL must, however, be validated in larger prospective studies.

In histopathological analyses performed in a disjoint autopsy cohort, the CSVD cases showed enhanced GFAP expression in astroglial cells and processes in the white matter around the lateral ventricular zone, whereas the upregulation of CHI3L1 was mainly observed in cell clusters located in the basal ganglia and thalamic nuclei. An additional autopsied case with mixed dementia—subcortical CSVD and moderate levels of AD-related changes (case #1)—showed the same expression pattern for GFAP and CHI3L1 in the subcortical white matter and nuclei, although an additional cortical expression of these astroglial markers was observed. An additional source of GFAP leakage to the CSF may be reactive astrocytes and gemistocytes found in stroke [21,38] and in cortical microinfarcts [35]. Furthermore, a common finding in the periventricular zone of elderly patients is venous collagenosis [47]. Whereas the exact mechanisms leading to an increase in the serum GFAP levels remain unresolved, it is tempting to speculate that GFAP released from astrocytes might be well-reflected in the serum, e.g., owing to disturbances in the blood–brain barrier and the close proximity of periventricular astrogliosis to the cerebrospinal fluid and the glymphatic system.

Despite the associations with disease severity and cognition, it is still not clear whether both serum NfL and serum GFAP are specific for CSVD in our cohort of elderly patients or whether both biomarkers may also reflect comorbid neurodegenerative pathologies such as impending Alzheimer’ disease [48,49]. Although patients with typical CSF patterns of Alzheimer’s disease were excluded from our study, there is a wide range of Amyloid-beta and total-Tau levels in moderate CSVD patients. In the present study, patients with higher mRS scores presented with significantly lower CSF levels of Amyloid-beta, albeit above the predefined cut-off required for the diagnosis of Alzheimer’s disease. This observation is in line with the observation that cognitive decline in elderly patients with CSVD is a heterogeneous process involving both white matter damage and the Amyloid-beta load and potentially also hippocampal atrophy [13,50,51,52]. Indeed, some studies even more explicitly suggested that CSVD may lead to dementia through interaction with Alzheimer’s pathology [53,54]. To further elucidate the role of neurodegeneration in CSVD, larger studies in independent cohorts with a broad approach, e.g., including Amyloid and Tau PET imaging to identify interactions of even subtle Alzheimer’s pathology, are needed [55,56].

Although MRI is the gold standard in the diagnosis and assessment of the CSVD burden, there are still limitations to the applicability and interpretation of MRIs. Besides the high costs and time expenditure, newer and more meaningful MRI techniques such as diffusion tensor imaging or white matter skeletonization require elaborate postprocessing, and different scanners and postprocessing methods lead to centre effects and difficulties in the reproducibility of CSVD imaging markers [12,57]. Furthermore, CSVD is a heterogeneous disease with respect to its aetiology, clinical presentation and course. Therefore, studies on CSVD usually examine different populations, from hereditary diseases to mixed diseases to selected patients, e.g., after recent lacunar infarction, whereas the most severely affected patients are usually not included [58]. It is thus difficult to get a holistic picture of the extent and impact of CSVD from a single feature such as WMH, and estimations of the total CSVD burden currently use dichotomised scales that fail to reflect the heterogeneity of different CSVD forms and pathologies. In addition, subvisible changes in normal-appearing white matter, subcortical microinfarcts and secondary neurodegeneration escape routine brain imaging, with the consequence that routine MRI imaging underestimates the true extent of CSVD. Therefore, new blood-based biomarkers that reflect the true extent of CSVD even in heterogeneous populations are urgently needed. These biomarkers would be of utmost importance to stage disease severity, monitor disease progression, identify subjects at risk of further cognitive decline and quantitatively measure the outcome and treatment effects in clinical trials [6,45,58].

Even though this is an explorative study with a limited number of patients, our findings underline the high potential of serum GFAP as a fluid biomarker in CSVD, as it shows a distinct correlation with clinical scores and neurocognitive function. Furthermore, this study provides insight into the expression profile of glial markers in CSVD patients by IHC, especially showing higher expression in the white matter zone in proximity to the ventricle. This might be an explanation for the good representation of the clinical severity of patients by the serum GFAP levels, as pathological processes that occur close the ventricle may result in GFAP increasingly draining from the CSF into the blood. Nevertheless, these findings remain to be validated in larger cohorts.

## 5. Conclusions

In this study, we were able to characterise a broad spectrum of CSF and serum biomarkers in CSVD patients. The findings further strengthen the utility of serum NfL in the diagnosis of CSVD and show that especially serum GFAP is a promising biomarker in CSVD. Analyses of GFAP and NfL in the serum open up a window of opportunity for consecutive analyses, and in addition to this, they are indicators of a more severe disease in patients with CSVD. However, these markers are not specific for CSVD and need to be integrated in the overall clinical evaluation. While the serum NfL levels have been studied longitudinally in CSVD patients before [3,12,13], it will be interesting to correlate the concentration of serum NfL to other serum biomarkers in longitudinal samples of CSVD patients and to compare the concentration of serum GFAP to clinical outcomes and matching controls. Especially, the correlation of GFAP and NfL with disease severity, and in the case of GFAP, the additional correlation with cognitive parameters may be further utilised in follow-up visits for monitoring the outcome of longitudinal clinical trials.

## Data Availability

The data presented in this study are available on request from the corresponding author. The data are not publicly available due to privacy and ethical restrictions.
